# Morpho‐Molecular Identification of Common Freshwater Loaches Collected From Different Ecosystems of Bangladesh

**DOI:** 10.1002/ece3.71559

**Published:** 2025-06-09

**Authors:** Md. Amdadul Haque, Saima Binte Hadi, Ayesha Akhter Sumona, Jonaira Rashid, Mohd Golam Quader Khan, Md. Samsul Alam

**Affiliations:** ^1^ Fisheries Biology and Genetics Bangladesh Agricultural University Mymensingh Bangladesh; ^2^ Fisheries Genetics Laboratory Bangladesh Fisheries Research Institute Mymensingh Bangladesh

**Keywords:** barcode gap, genetic divergence, loach, phylogenetic tree, species delimitation

## Abstract

The biodiversity of hill stream fishes, especially loaches, in Bangladesh is declining, despite limited exploration. Conservation strategies can flourish through the effective identification of species. Ambiguous loaches were collected to resolve taxonomic confusion. Morphology was analyzed through morphometric and meristic traits, with mitochondrial COI and nuclear RAG1 genes as molecular markers. Sixteen morphotypes are found in 60 specimens, and the molecular study confirmed the existence of seven species. Furthermore, the species delimitation method ASAP suggests seven species according to the best partition. A low level of intraspecific diversity is found in the RAG1 gene analysis compared with the COI gene. The lowest interspecific genetic divergence (3.7%) was observed between *Botia lohachata* and *Botia rostrata* , whereas *Canthophrys gongota* showed the highest interspecific genetic divergence (24.45%) with *B. lohachata.* A potential barcoding gap of 2.52% in the COI gene is typically the threshold for distinguishing intra‐species from inter‐species comparisons. In the maximum likelihood phylogenetic tree of both COI and RAG1 gene sequences, the species are divided into distinct clades with high bootstrap support, and specimens within the same species do not converge. Morphology, along with the molecular data of this study, will strengthen conservation efforts for loaches.

## Graphical abstract

Taxonomic identification of seven morphologically ambiguous freshwater loach species of Bangladesh has been confirmed through morphological study as well as analyzing mitochondrial and nuclear DNA sequences. A relatively low level of intraspecific genetic diversity has been found in the nuclear RAG1 gene compared to the mitochondrial COI gene of the loaches.

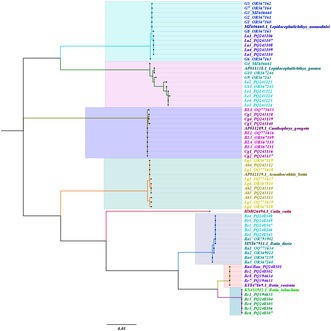

## 1 Introduction

Bangladesh is a deltaic country with 700 rivers enriched with a wide range of aquatic diversity, comprising 253 freshwater fish species (IUCN Bangladesh 2015). Inland water systems in this ecozone provide a secure haven for indigenous living systems, including ichthyofauna. Among freshwater species, more than 143 species have been identified as small indigenous species (SIS). Fish that reach a mature or adult size of 25 cm or less are known as SIS (Felts et al. 1996). With a flat topography, hilly areas cover 12% of the country and are concentrated in the northeast and southeast regions. Distinct habitats with sandy or gravelly bottoms and clear standing water create a vast aquatic biodiversity. About 82 fish species were identified from this habitat (Ahmed et al. 2013). Hill stream fishes belonging to the families Psilorhynchidae, Cobitidae, and Balitoridae, included in SIS, are commonly known as loaches.

At least 24 species of loaches belonging to three families and nine genera have been identified in Bangladesh: Cobitidae (12 species), Balitoridae (nine species), and Psilorhynchidae (three species) (Habib et al. 2023; Hossain et al. 2012). Once upon a time, loaches were a highly abundant group of freshwater fish species in Bangladesh. Most of these species inhabit the hill streams of Sylhet, Dinajpur, and Mymensingh. However, according to the IUCN Bangladesh, the current biodiversity status of the 24 loaches shows that two are critically endangered, five are endangered, two are vulnerable, five are data deficient, six are of least concern, and four are not threatened. Akand et al. (2015) observed that the availability of fish was comparatively higher in the winter season than the rest of the year, and *Lepidocephalichthys guntea* , *Canthophrys gongota*, and *Acanthocobitis botia* were more available among the observed loach species than others. Loaches have more complex biodiversity than other fish. The taxonomy and systematics of these fish are still controversial due to minor morphological differences. Loaches that inhabit similar environments tend to develop similar characteristics. Therefore, it is probable that the great resemblance among various species is due to their shared environment (Goswami et al. 2022).

Moreover, the local names of the different species of loaches are similar, such as *L. guntea* and *Lepidocephalichthys annandalei* , which are locally known as Gutum, while *Botia dario* , *Botia lohachata*, and *Botia rostrata* are referred to as Rani. Inadequate studies about loaches make them ambiguous. Whereas some species of loaches are now under induced breeding, accurate identification is essential for the induced breeding of loaches to prevent crossbreeding between species.

Accurately identifying fish is crucial for conservation management, making it a key responsibility for taxonomists and ichthyologists (Shen et al. 2016). Morphometric and meristic analyses serve as the primary foundation for fish classification and identification (Triantafyllidis et al. 2011). Classical taxonomy has significantly contributed to species classification based on morphological characteristics. However, due to the plasticity of morphology, traditional taxonomy cannot accurately differentiate all species, especially similar and closely related ones (Pigliucci 2005). Loaches exhibit several morphologically distinct forms during their lives, yet it can be difficult to distinguish them at a glance. Comparatively few morphological characters have been used for taxonomic studies, resulting in many unresolved puzzles (Bohlen et al. 2011). DNA‐based identification techniques provide a significant analytical complement or an alternative (Teletchea 2009). Hebert et al. (2003) proposed using 650‐bp segments from the mitochondrial gene cytochrome oxidase subunit 1 (COI) to detect and identify unknown species. RAG1 is a prominent nuclear marker for phylogenetic analysis due to its ability to reveal links between and within vertebrate lineages (Chiari et al. 2009). In recent years, RAG1 DNA sequences have been employed for phylogenetic analysis across different taxonomic levels.

Furthermore, DNA barcodes can successfully identify fish species and reveal intra‐ or interspecific variation among species (Ward et al. 2009). However, nuclear mitochondrial pseudogenes raise questions about DNA barcodes (Song et al. 2008). The database shows a discrepancy due to mismatches with species that have been mislabeled or misidentified.

Historically, loaches were abundant in the ponds, rivers, and streams of Bangladesh; however, they are now rarely found in these habitats. Limited research on loaches has led to confusion, as many morphological studies have inaccurately described these fish. The study focused solely on morphological variations during sampling to determine whether the specimens belonged to the same species. As a result, this study critically examined loaches, collecting morphologically ambiguous specimens to identify them at the species level using both morphological and molecular approaches.

## 2 Materials and Methods

### 2.1 Sampling Area

**FIGURE 1:**
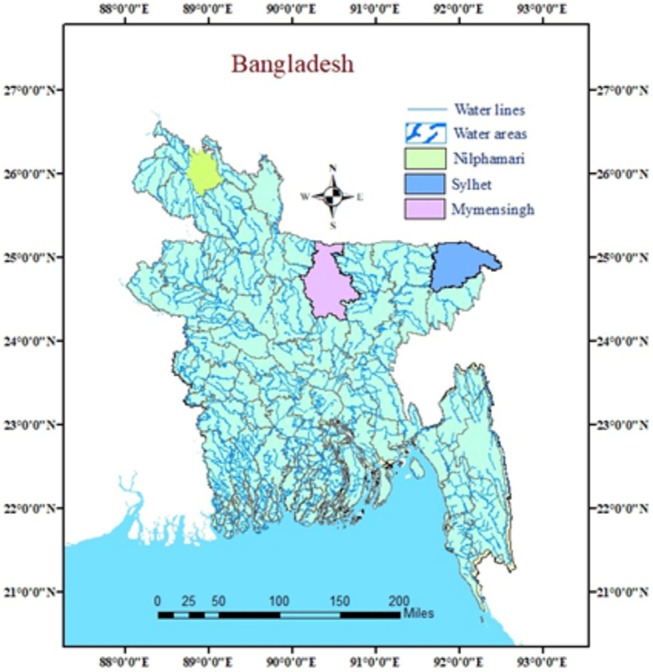
Sampling sites. Specimens were collected from Nilphamari, Mymensingh, and Sylhet.

This research aimed to study the morphological and molecular variations among the loaches. In total, 60 specimens were collected from different locations in Mymensingh, Saidpur, Nilphamari, and Sylhet (Figure 1). The specimens were nearly uniform in size for each species. The specimens were stored in an ice box, with each sample contained in a unique sterilized polythene zipper bag that included specific information. The ice box was taken to the Department of Fisheries Biology and Genetics Laboratory at Bangladesh Agricultural University in Mymensingh. The investigation was carried out on deceased fish.

### 2.2 Morphological Approach for Identifying the Specimens

**FIGURE 2:**
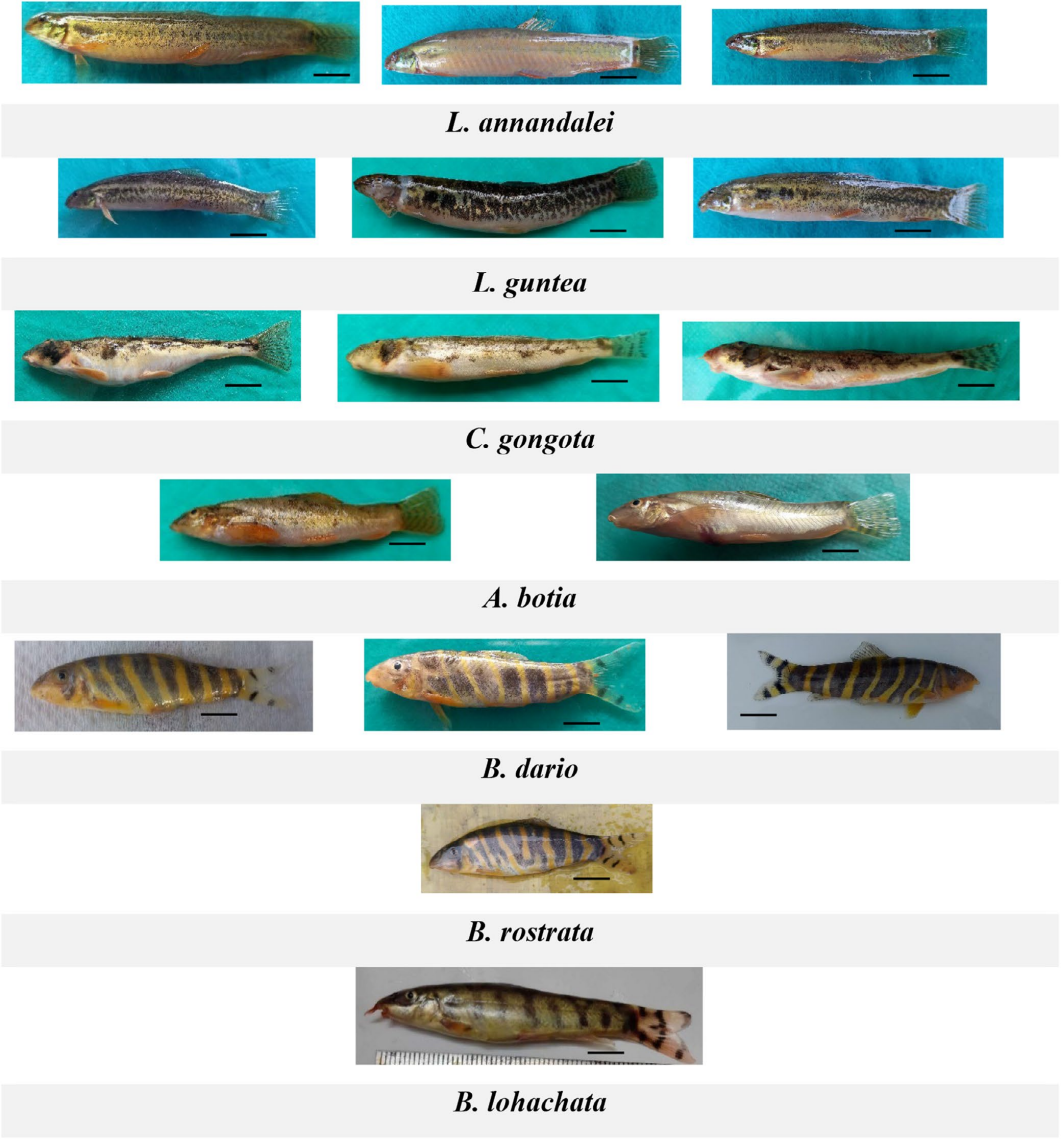
Morphotypes of the collected specimens. Three morphotypes were found in the case of *L. annandalei, L. guntea, C. gongota*, and *B. dario* , and two in the case of *A. botia*. A single morphotype was found in *B. rostrata* and *B. lohachata* specimens. Scale bar 10 cm.

The study categorized specimens based on morphological characteristics (Figure 2), including total length, standard length, head length, pectoral fin length, and pelvic fin length, and counted scales and fin rays, according to the reference book “Freshwater fishes of Bangladesh” (Rahman 2005). Morphological correlation was determined through a principal component analysis (PCA) plot using PAST software (Hammer 2001).

### 2.3 Molecular Methods to Identify the Specimens

#### 2.3.1 DNA Extraction

The total DNA was isolated from the fin tissue of each individual using proteinase K digestion, phenol–chloroform–isoamyl alcohol purification, and the ethanol precipitation method as described by Islam and Alam (2004). Agarose (1.2%) gel electrophoresis was used to analyze the DNA samples. The DNA of fish tissue samples was quantified through absorbance reading using the Nanodrop spectrophotometer (NanoDrop one 3000; USA).

#### 2.3.2 PCR Amplification

The primer set (Forward primer Fish F1: TCAACCAACCACAAAGACATTGGCAC and Reverse primer Fish R1: TAGACTTCTGGGTGGCCAAAGAATCA) (Ward et al. 2005) was used for PCR amplification of the COI gene, and Forward primer RAG1: AGCTGTAGTCAGTAYCACAARATG and Reverse primer RAGm‐RV1: TCCTGRAAGATYTTGTAGAA (Šlechtová et al. 2007) were used for PCR amplification of the RAG1 gene. A 50 μL reaction mixture was prepared for PCR amplification of each specimen. For the COI gene, the thermal program was set for an initial denaturation at 94°C for 3 min followed by 35 cycles each comprising denaturation at 94°C for 45 s, annealing at 54°C for 1 min, extension at 72°C for 1 min 30 s, and final extension at 72°C for 7 min. For the RAG1 gene, the gradient temperature was set at annealing 48°C–55°C for 45 s and the other temperature profile was the same. The partial amplification of two genes (650 bp for COI and 800 bp for RAG1) was confirmed by 1.2% agarose gel electrophoresis. Following the manufacturer's instructions, the BigDye Terminator Cycle Sequencing Kit (Applied Biosystems) was used to directly sequence the purified PCR products (free of salts and protein contaminants) in the Applied Biosystems 3500 genetic analyzer. Sequencing was done at Apical Scientific Sdn Bhd, Malaysia.

#### 2.3.3 Sequence Data Analysis

Bio‐Edits software was used to edit nucleotide sequences (Hall 1999). Low‐quality chromatograms were deleted, and clear peak chromatograms were selected and copied to Microsoft Word. The National Centre for Biotechnology Information (NCBI) provides an online tool called the Basic Local Alignment Search Tool (BLAST) to analyze the homology of mtDNA COI gene nucleotide sequences with the GenBank data. The multiple sequence alignment was done with ClustalW (Thompson et al. 1994), and the default parameters of MEGA‐X were set (Kumar et al. 2018). In the case of pairwise and multiple alignment, the gap opening penalty was 15, and the gap extension penalty was 6.6. MEGA‐X estimated pairwise genetic distance for the sequences using the maximum composite likelihood approach (Tamura et al. 2004) and the Kimura‐2 Parameter model (Kimura 1980). The phylogenetic trees of specimens were reconstructed using the Maximum likelihood method (Saitou and Nei 1987) with the Kimura‐2 Parameter model (Kimura 1980) and the maximum composite likelihood method used with a bootstrap value of 100 (Felsenstein 1985) in the estimation of evolutionary distances of the sample in MEGA‐X. The phylogenetic tree was modified through FigTree v1. 3.1 (Rambaut and Drummond 2010). Haplotype diversity, gene flow, genetic differentiation, and molecular variance analysis were performed using DnaSP software (Rozas et al. 2017). A haplotype network was constructed using NETWORK Ver. 10.2 software and median‐joining network protocol (Bandelt et al. 1999). The Automated Barcode Gap Discovery (ABGD) analysis was conducted using the Jukes‐Cantor (JC69) model in https://bioinfo.mnhn.fr/abi/public/abgd/abgdold.html, a web‐based tool (Puillandre et al. 2012). The Assemble Species by Automatic Partitioning (ASAP) study (Puillandre et al. 2021) used the web interface with default parameters at https://bioinfo.mnhn.fr/abi/public/asap. The geographic location of the sampling area was shown on the map using ArcGIS 10.8 (ESRI 2011).

## 3 Result

### 3.1 Morphological Identification

**FIGURE 3:**
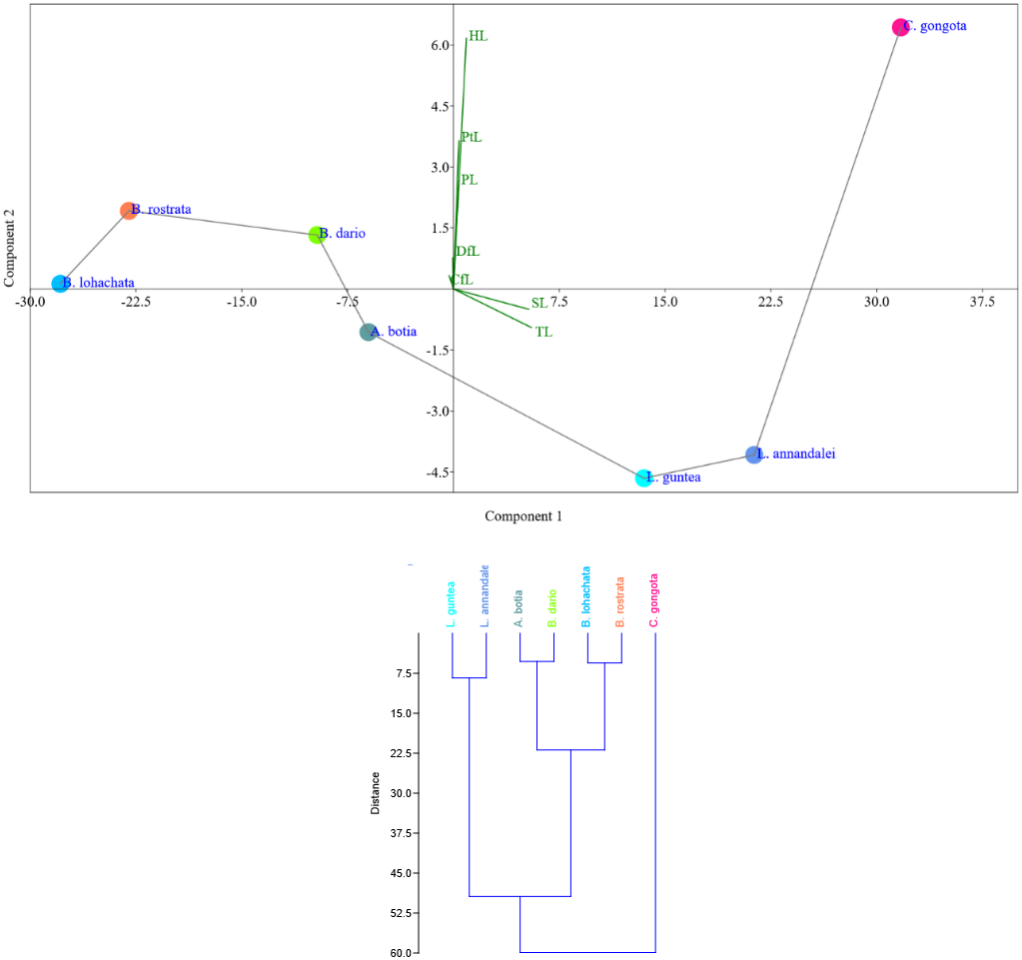
PCA plots of morphometric characters of the collected specimens and dendrogram based on morphometry.

The study examined morphometric and meristic traits to identify 60 fish specimens, belonging to the Cobitidae and Balitoridae families (Table 1). Three genera were identified from the Cobitidae family, while one genus was identified from the Balitoridae family. During the study, several coloration patterns were observed, resulting in a total of 16 morphotypes among the 60 specimens. All morphotypes exhibited variations in body coloration and banding patterns, while other morphological characteristics were almost identical. The species level remained ambiguous due to morphological complexity. In the PCA plot, *L. guntea, L. annandalei*, and *A. botia* were in component 1, but *L. guntea* and *L. annandalei* were highly correlated. *C. gongota, B. dario, B. lohachata*, and *B. rostrata* were located in component 2, where *B. dario, B. lohachata*, and *B. rostrata* showed high correlation (Figure 3). In the dendrogram, *A. botia* and *B. dario* formed a cluster together.

**TABLE 1:** Morphological characteristics of specimens.

Morphological characters	*L. guntea*	*L. annandalei*	*C. gongota*	*A. botia*	*B. dario*	*B. lohachata*	*B. rostrata*
Pigmentation Pattern on the body side	Color variable, a light band (>) dorsal to ventral from snout to caudal, and many discontinuous round blotches on the lateral line.	Light brown to silvery color and irregular brown blotches along the lateral line.	Irregular band along the lateral line and 5–6 dark blotches on the body	Irregular dark band which ends in a dark spot just under the lateral line.	Seven black bands on the brown body, upper to lower side of the body, and 1 band on the head	Body color yellow or yellowish with 4 Y‐shaped transverse bands on the body runs from dorsal to ventral, and transverse bars at the center of every two bands	Body color is yellowish to brown and has five body bars. Usually paired vertical bars on the body, often with less‐vivid base body color.
Black center on the caudal peduncle	A dark center on the caudal base	A clear dark spot in the caudal fin.	No black center on the caudal base	A dark center on the upper caudal base.	No black center on the caudal fin.	An obscure vertical bar on the caudal base	A medium to dark vertical bar on the caudal base
Bands on the caudal fin	Numerous spots were found throughout the caudal fin.	4–5 > – shaped bands on the caudal fin	4 obscure band on the caudal fin	5–7 > – shaped bands, which were formed of spots	Two black bands, where one is continuous and the other is discontinuous	2–3 cross‐shaped bands.	Caudal fins develop a bright orange/golden color and two dark bands on the caudal fin.
Fin formula	D. 7–8 (2/5–6); P_1_. 8; P_2_. 8; A.7 (2/5)	D. 8 (1/7); P_1_. 8; P_2_. 6–7; A.7 (2/5)	D. 10 (2/8); P_1_. 10; P_2_.8; A.7 (2/5)	D. 12–13 (2/5–6); P_1_. 10; P_2_. 8; A.6 (1/5)	D. 11 (2/9); P_1_. 14; P_2_. 8; A.7 (2/5)	D. 10–11 (1/9); P_1_. 14; P_2_. 8; A. 7 (1/6)	D. 11 (2/9); P_1_. 14; P_2_. 8; A.7 (2/5)

### 3.2 Molecular Identification

The BLAST search results showed an average homology of 99.31% to 100%, with query coverage ranging from 98% to 100%. The average similarities with the reference sequences of *L. annandalei, L. guntea, B. dario, C. gongota, A. botia, B. lohachata*, and *B. rostrata* were 99.95%, 99.31%, 99.59%, 99.69%, 99.69%, 100%, and 100%, respectively. The average values for T, A, C, and G in the sequence were 29.9% ± 1.15%, 24.5% ± 0.91%, 28.2% ± 1.21%, and 17.4% ± 0.67%, respectively. The A + T content of the COI gene was 54.4%, and the G + C content was 45.6%. The A + T content of the RAG1 gene was 49.2%, and the G + C content was 50.8%. All sequences of the COI and RAG1 genes have been deposited in GenBank, and each sequence has been provided a GenBank accession number (Table 2).

**TABLE 2:** GenBank accession number and conservation status of all the inferred specimens included in the molecular phylogeny.

SL no.	Voucher ID	Family	Scientific name	GenBank accession no. COI	GenBank accession no. RAG1	Conservation status
1	G1	Cobitidae	*L. annandalei*	OR567160	PQ409353	Vulnerable
2	G2		*L. annandalei*	OR567161	PQ409354	
3	G3		*L. annandalei*	MZ606660	—	
4	G5		*L. annandalei*	OR567162	—	
5	G6		*L. annandalei*	OR567163	PQ282203	
6	G7		*L. annandalei*	OR567164	—	
7	G8		*L. annandalei*	OR567165	PQ282204	
8	La1		*L. annandalei*	PQ243106	PQ282207	
9	La2		*L. annandalei*	PQ243107	PQ282208	
10	La3		*L. annandalei*	PQ243108	PQ282209	
11	La4		*L. annandalei*	PQ243109	PQ282210	
12	La5		*L. annandalei*	PQ243110	PQ282221	
13	G4	Cobitidae	*L. guntea*	MZ606661	—	Least concern
14	G9		*L. guntea*	OR567243	—	
15	G10		*L. guntea*	OR567244	—	
16	G11		*L. guntea*	OR567245	—	
17	Le1		*L. guntea*	PQ243122	PQ464095	
18	Le2		*L. guntea*	PQ243123	PQ464096	
19	Le3		*L. guntea*	PQ243124	PQ464097	
20	Le4		*L. guntea*	PQ243125	PQ464098	
21	Le5		*L. guntea*	PQ243126	PQ464099	
22	Ra1	Cobitidae	*B. dario*	OQ773634	—	Endangered
23	Ra2		*B. dario*	OR569021	—	
24	Ra3		*B. dario*	OR793902	—	
25	Ra4		*B. dario*	OR567259	—	
26	Ra5		*B. dario*	OR567260	—	
27	Br1		*B. dario*	PQ248545	—	
28	Br2		*B. dario*	PQ248546	—	
29	Br3		*B. dario*	PQ248547	—	
30	Br4		*B. dario*	PQ248548	—	
31	Br5		*B. dario*	PQ248549	—	
32	BL1	Cobitidae	*C. gongota*	OQ773635	PQ317756	Near threatened
33	BL2		*C. gongota*	OQ773636	PQ317757	
34	BL3		*C. gongota*	OR567109	PQ317758	
35	BL4		*C. gongota*	OR567110	—	
36	BL5		*C. gongota*	OR567111	PQ317759	
37	Cg1		*C. gongota*	PQ243136	PQ317760	
38	Cg2		*C. gongota*	PQ243137	PQ317761	
39	Cg3		*C. gongota*	PQ243138	PQ317762	
40	Cg4		*C. gongota*	PQ243139	PQ317763	
41	Cg5		*C. gongota*	PQ243140	PQ317764	
42	Lg1	Balitoridae	*A. botia*	OQ773637	PQ282211	Least concern
43	Lg2		*A. botia*	OQ773638	PQ282212	
44	Lg3		*A. botia*	OQ773639	PQ282213	
45	Lg4		*A. botia*	OR567118	PQ282214	
46	Lg5		*A. botia*	OR567119	PQ282215	
47	Lg6		*A. botia*	OR567120	—	
48	Ab1		*A. botia*	—	PQ282216	
49	Ab2		*A. botia*	PQ243310	PQ282217	
50	Ab3		*A. botia*	PQ243311	PQ282218	
51	Ab4		*A. botia*	PQ243312	PQ282219	
52	Ab5		*A. botia*	PQ243313	PQ282220	
53	Bc1	Cobitidae	*B. lohachata*	PQ394635	—	Endangered
54	Bc3		*B. lohachata*	PQ248504	—	
55	Bc4		*B. lohachata*	PQ248505	—	
56	Bc5		*B. lohachata*	PQ248506	—	
57	Bc6		*B. lohachata*	PQ248507	—	
58	Bc2	Cobitidae	*B. rostrata*	PQ248502	—	Data deficient
59	Ra4.Bau		*B. rostrata*	PQ248501	—	
60	Bc7		*B. rostrata*	PQ394633	—	
61	Rc8		*B. rostrata*	PQ394634	—	

#### 3.2.1 Genetic Diversity and Divergence Analysis of the COI and RAG1 Genes

**FIGURE 4:**
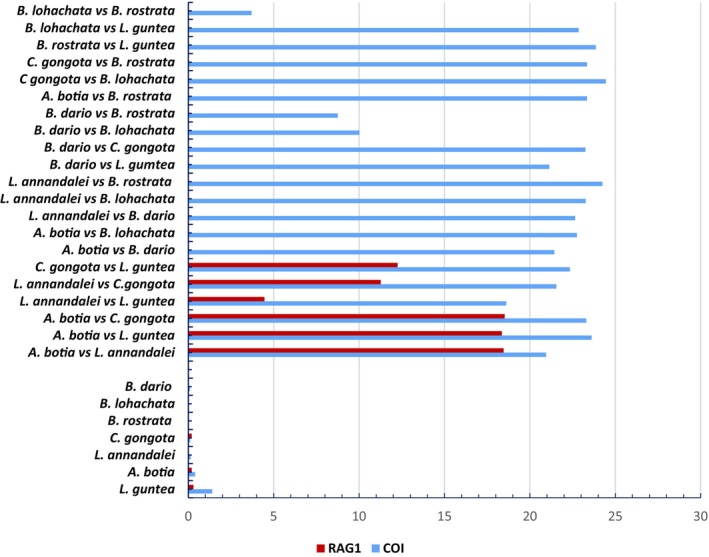
Mean intra‐ and interspecific genetic distance of the mitochondrial COI and nuclear RAG1 genes of the collected specimens.

The COI data set consisted of 422 invariable (monomorphic sites) and 215 variable (polymorphic) sites, with 4 being singleton variable sites and 211 being parsimony informative sites. The overall haplotype diversity (Hd) was 0.9559. The haplotype diversity of *L. guntea* was found to be 0.91667, and the lowest haplotype diversity was found to be zero in the *B. lohachata* specimen. The overall nucleotide diversity (Pi) was 0.15798. In this study, pairwise genetic distance analysis was conducted using the K2P distance model in MEGA X software involving 60 nucleotide sequences. Genetic distance ranged from 0.0000 to 0.2621, and the overall mean genetic distance was 0.19 ± 0.04. Within the *L. annandalei* species, the genetic diversity ranged from 0.0000 to 0.0031. Within the *L. guntea* species, the genetic diversity ranged from 0.000 to 0.0204. The highest genetic diversity was observed between G4 and Le3, which was 0.0189. In the case of the *B. dario* species, the genetic diversity ranged from 0.000 to 0.0031. Genetic diversity in the *C. gongota* species ranged from 0.0015 to 0.0031. Within the *A. botia* species, the genetic diversity ranged from 0.000 to 0.0078. No genetic diversity was found within *B. lohachata* and *B. rostrata*. The mean genetic diversity within *L. annandalei* , *L. guntea* , *C. gongota*, *A. botia* , and *B. dario* was observed to be 0.14%, 1.4%, 0.11%, 0.45%, and 0.1%, respectively. The highest mean genetic diversity was observed in the case of *L. guntea* at 1.4%. The mean genetic divergence between *L. annandalei* and *L. guntea* , *L. annandalei* and *B. dario, L. annandalei* and *C. gongota*, and *L. annandalei* and *A. botia* was observed at 18.61%, 22.65%, 21.55%, and 20.95%, respectively. Between *L. guntea* and *B. dario* , *L. guntea* and *C. gongota*, *L. guntea* and *A. botia* , *B. dario* and *C. gongota, B. dario* and *A. botia, C. gongota* and *A. botia* , *B. lohachata* and *L. annandalei* , *B. lohachata* and *L. guntea* , *B. lohachata* and *C. gongota*, *B. lohachata* and *A. botia* , *B. lohachata and B. dario*, *B. rostrata* and *L. annandalei* , *B. rostrata* and *L. guntea* , *B. rostrata* and *C. gongota*, *B. rostrata* and *A. botia* , *B. rostrata* and *B. dario* , *and B. lohachata* and *B. rostrata* , the mean genetic divergence was observed at 21.14%, 22.34%, 23.61%, 23.25%, 21.43%, 23.31%, 23.27%, 22.85%, 24.45%, 22.75%, 10.02%, 24.25%, 23.86%, 23.34%, 23.34%, 8.75%, and 3.7%, respectively (Figure 4).

The RAG1 data set had 603 invariable (monomorphic) sites and 177 variable (polymorphic) sites. Among the variable sites, there were 3 singletons and 174 parsimony‐informative. The overall haplotype and nucleotide diversity were 0.89015 and 0.10207. In the case of four species, genetic diversity within *L. annandalei, L. guntea*, *C. gongota*, and *A. botia* was observed to be 0%, 0.3%, 0.2%, and 0.2%, respectively. The mean genetic divergence between *L. annandalei* and *L. guntea* , *L. annandalei* and *C. gongota, L. annandalei* and *A. botia, L. guntea* and *C. gongota*, *L. guntea* and *A. botia* , and *C. gongota* and *A. botia* were observed to be 4.46%, 11.27%, 18.46%, 12.26%, 18.36%, and 18.52%, respectively.

**FIGURE 5:**
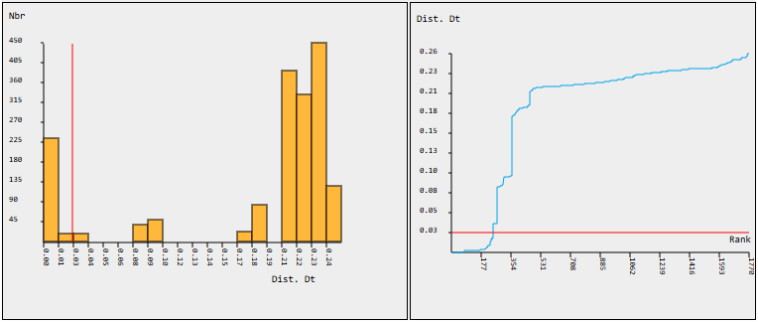
The histogram shows the distribution of pairwise genetic divergences (model‐Kimura 80) of collected loach specimens, and the curve suggests the ranked pairwise differences. The red line shows the cumulative frequency of the distance values of delimited species.

According to ABGD analysis, there was a putative barcoding gap at the level of 2.52% variations in the COI gene, typically the threshold for the distinction between intra‐species and inter‐species comparisons (Figure 5). Another putative gap was found at the level of 8% variations in the COI gene.

#### 3.2.2 Phylogenetic Tree and Species Delimitation

**FIGURE 6:**
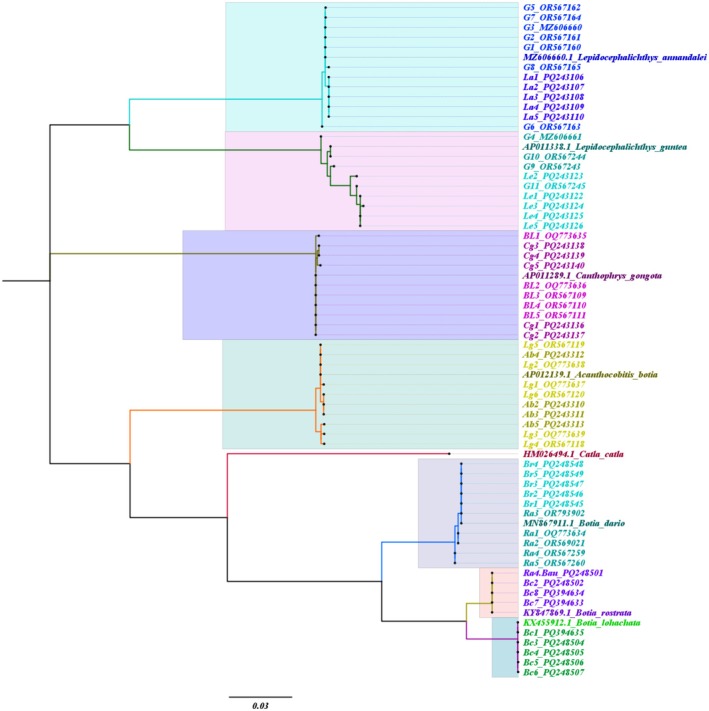
Molecular phylogenetic tree of sample sequences of the COI gene reconstructed by the Maximum likelihood method (K2P model) with 1000 bootstrap values. *Catla catla* was used as an out‐group. Each highlight represents an individual species.

**FIGURE 7:**
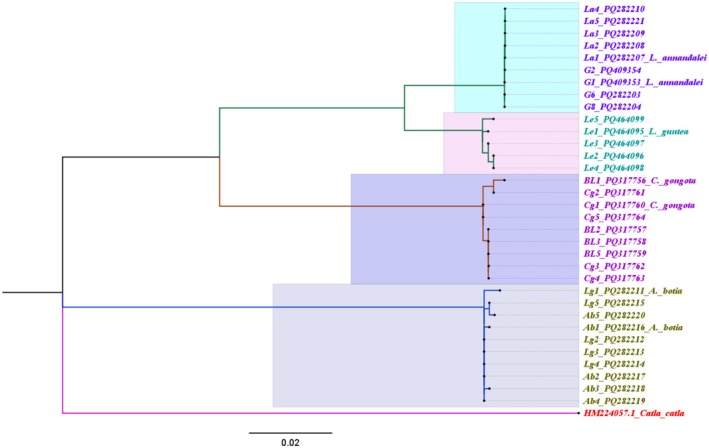
Molecular phylogenetic tree of sample sequences of the RAG1 gene reconstructed by the maximum likelihood method (K2P model) with 1000 bootstrap values. Each highlight represents an individual species.

**FIGURE 8:**
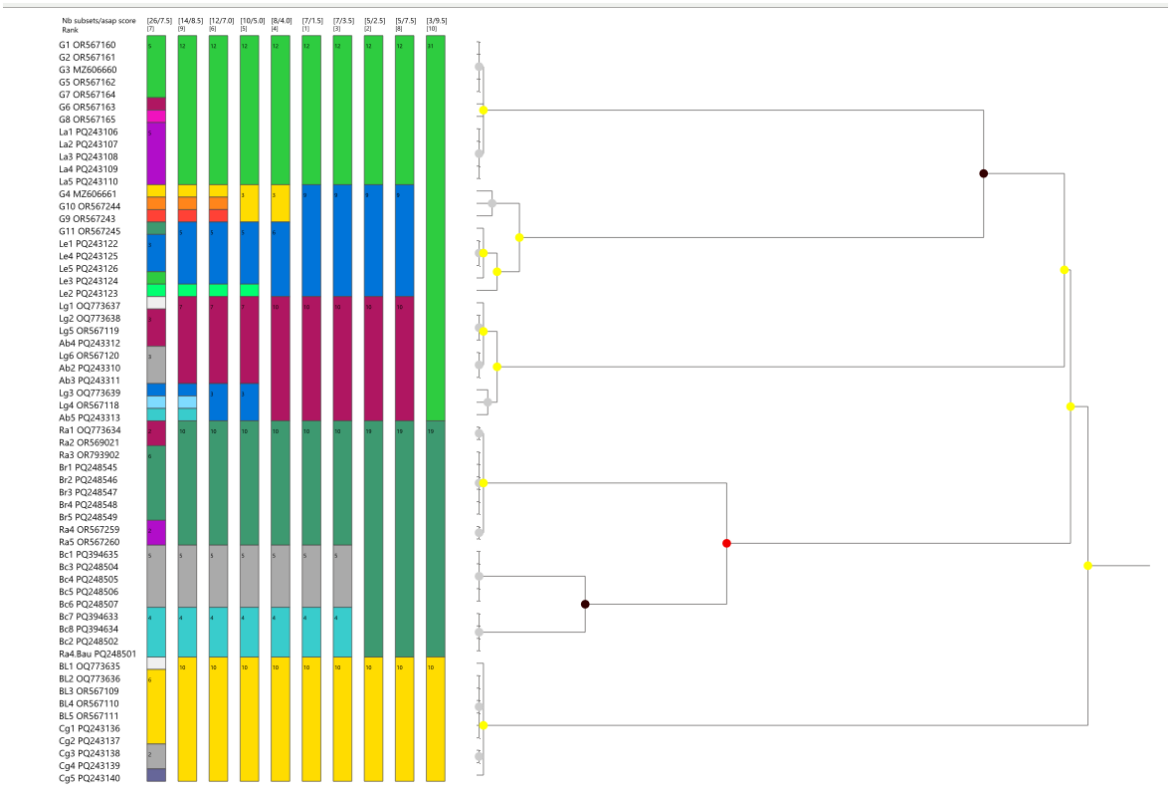
ASAP analysis of the COI gene for species delimitation, where the colorful bar indicates the number of species, and each bar contains the number of individuals.

The phylogenetic trees were reconstructed based on the COI and RAG1 gene sequences of collected specimens using the maximum likelihood method. In the COI tree, each sample was clustered with its reference genome (Figure 6). *Catla catla* was used as an out‐group in the phylogenetic tree. Seven primary and 26 secondary molecular taxonomy units (MOTUs) were found in the COI gene analysis. In the phylogenetic tree, seven major clades with strong bootstrap support represent the number of putative species. In the RAG1 tree, sequences from four species were used, and all sequences were clustered according to each species (Figure 7). Four major clades, each supported by strong bootstrap values, represent the number of proposed species. In the ASAP analysis of the COI gene, the best partition was found at 1.5 ASAP score (lowest) with a 0.0252 threshold distance, where the number of subsets was seven, representing the primary species hypotheses (Figure 8).

#### 3.2.3 Haplotype Network

**FIGURE 9:**
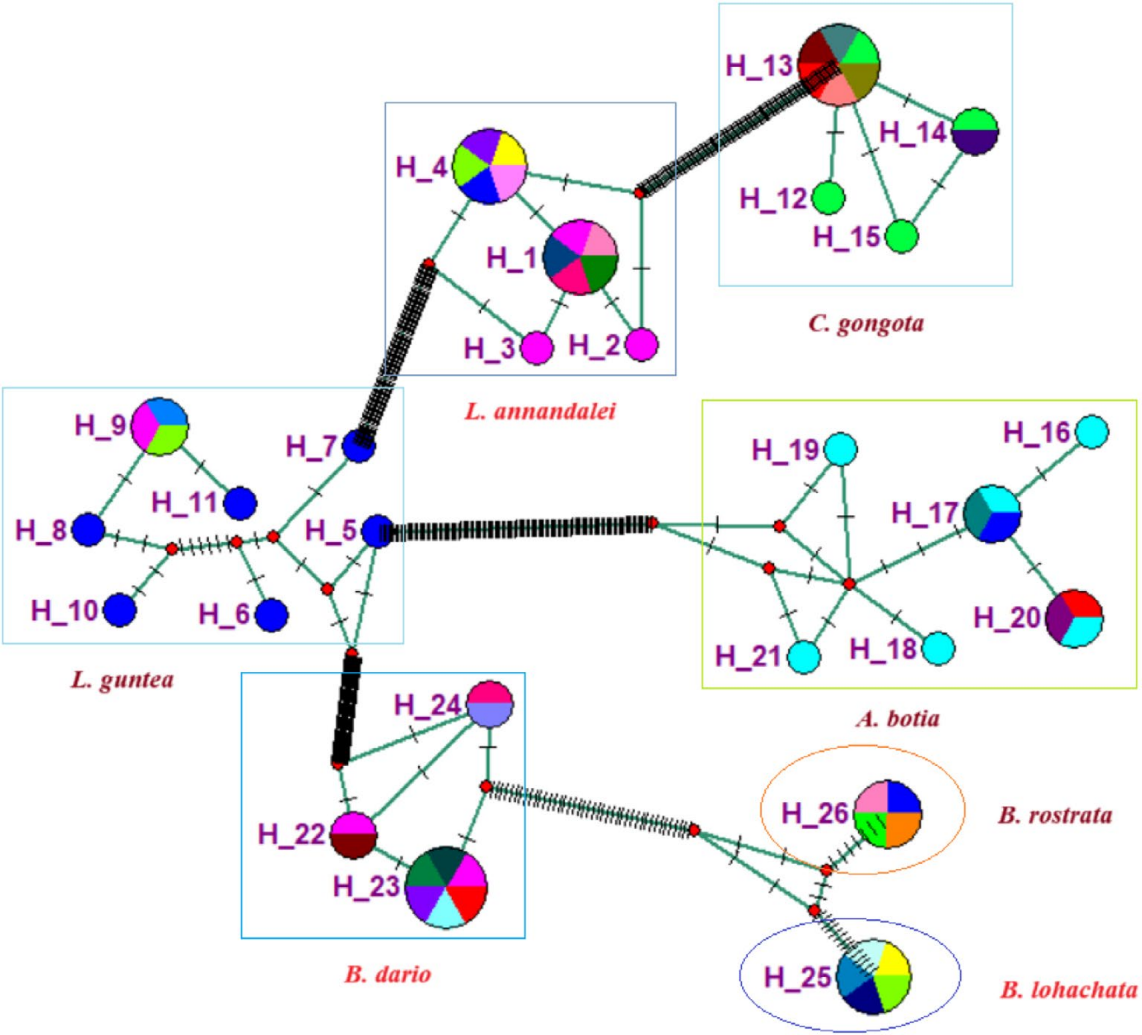
Median‐joining haplotype network of the COI gene sequences, where the color circle represents the haplotypes denoted by H. The bar across branches indicates the number of mutations that separate two haplotypes.

The median‐joining haplotype network of the collected specimens is shown in Figure 9. In total, 26 haplotypes were found in the COI gene sequences. The highest seven haplotypes were found in *A. botia*, and a single haplotype was found each in *B. lohachata* and *B. rostrata*. The overall 14 unique haplotypes were found in the analysis.

## 4 Discussion

External physical features, including body shape, coloration, size, the number of scales, the number of fins, type and quantity of fin rays, and the relative measurements of body parts, have been conventionally used to distinguish between different fish species (Strauss and Bond 1990). Morphological identification was performed using significant features such as the pigmentation pattern on the side of the body, the black center on the caudal peduncle, and the bands on the caudal fin of loaches (Table 1). The body‐side pigmentation pattern has traditionally been regarded as the most crucial trait for classifying Cobitid species (Chen et al. 2015). The color pattern of the dark‐to‐dark brown band on the body side of the fish showed significant differences. A black center on the caudal peduncle was found in the cases of *L. annandalei* , *L. guntea* , and *A. botia* , while no black center was found in the cases of *C. gongota*, *B. dario* , *B. lohachata* , and *B. rostrata* specimens. The pattern of bands on the caudal fin showed significant differences, although variation was found in the same species of *L. guntea* . In this study, the numbers of dorsal, anal, and pectoral fin rays were observed to be similar to those obtained previously by Rahman (2005).

The number of fin rays was very similar in *L. annandalei* and *L. guntea* . The pectoral and pelvic fins of *L. guntea* were found to be almost equal in length. In the case of *L. annandalei* , *L. guntea* , *C. gongota*, and *B. dario* , three morphotypes were found where body‐side coloration and banding pattern slightly differed. The G11 specimen of *L. guntea* looked different from the other three. As for *A. botia* , two morphotypes were found, and the appearances of Lg3 and Lg4 specimens differed. The morphotypes were not limited to specific regions; the same morphotype was observed in different geographical areas. Habitat differences may cause intraspecific morphotypes. The muddy and sandy bottom could influence the body coloration, as the specimens were collected from different ecological zones.

According to the blast analysis, mtDNA COI sequences of all species in this research had average homology ranging from 99.31% to 100% with the reference sequences, which was satisfactory. Notably, no morphotypes crossed the species boundaries. Haque et al. (2018) showed that the sequences of known specimens of the two species ( *Osteobrama cotio* and *Esomus danricus* ) that are accessible in the database shared significant levels of similarity (98–100%) with the results. This study first deposited nucleotide sequences of the COI and RAG1 genes of *L. annandalei* in GenBank. In this study, the deficiency of the nucleotide G was observed in all mtDNA COI sequences (average 17.4% ± 0.67%) compared to the other three bases (on average *T* = 29.9% ± 1.15%, *A* = 24.5% ± 0.91%, *C* = 28.2% ± 1.21%). The *A* + *T* content (54.4%) was higher than the *G* + *C* content (45.6%), which was similar to Pacific fishes of Canada (Steinke et al. 2009).

Genetic diversity markers include haplotype and nucleotide diversity, indicating genetic variation within a group (Falush et al. 2003; Liu 2017). In this study, the overall haplotype diversity (Hd) was 0.9559. When compared to many other species, the haplotype diversity ranges from 0.75 to 0.92, which is high (De Jong et al. 2011). So, haplotype diversity showed that the loach species in Bangladesh had a high level of haplotype heterogeneity. Pandey et al. (2020) found a Hd of 0.975 for the entire dataset, indicating high genetic diversity in the Ranganadi River. The haplotype diversity of *L. guntea* was found to be 0.91667, and the lowest haplotype diversity was found to be zero in the *B. lohachata* specimen. *L. guntea* is genetically more diverse, but there is a lack of diversity in *C. gongota*. The overall nucleotide diversity (Pi) was 0.1579. Different families had different nucleotide diversity, which varied by 17% for the family Cyprinidae (Ali et al. 2020). High haplotype diversity and low nucleotide diversity indicate only minor variations across haplotypes (De Jong et al. 2011). High haplotype diversity and nucleotide diversity indicate significant differences among haplotypes. The COI gene contains 33.33% polymorphic sites, comparatively higher than the nuclear RAG1 gene (22.69%). Perdices et al. (2016) found 22.4% of informatics sites in the RAG1 gene of the European spined loaches.

In general, intraspecific DNA barcode variation is significantly less than interspecific DNA barcode variation (Savolainen et al. 2005). In this study, genetic distance ranged from 0.0000 to 0.2621. Guo and Zhang (2021) found 0.1% to 0.8% intraspecific genetic distance in the case of *Leptobotia* species. Sheraliev and Peng (2021) found that the average intraspecific K2P distance was 0.22% in their study. Other studies showed low‐level average intraspecific diversity, such as 0.39% (Ward et al. 2005), 0.22% (Zangl et al. 2022), and 0.40% (Lakra et al. 2016). In this study, comparatively lower intraspecific genetic diversity was observed in *L. annandalei* , *C. gongota*, and *A. botia*, and no genetic diversity was observed in *B. lohachata* and *B. rostrata* . In the case of *L. guntea* , *the* mean intraspecific genetic diversity was comparatively higher at 1.4%. No specimens exceed the threshold range of 2%, as described by Hebert and Gregory (2005); in most cases, intraspecific divergences are less than 1% and almost rarely exceed 2%. In ABGD analysis, a barcode gap was also found between 2% to 3%. Increased divergence within the *L. guntea* species may reflect geographical isolation. As mtDNA is maternally inherited, gene flow within a population and interpopulation may affect the diversity of COI within a species. A low level of intraspecific diversity was found in nuclear RAG1 gene analysis compared with the mitochondrial COI gene.

The lowest interspecific genetic divergence (3.7%) was observed between *B. lohachata* and *B. rostrata*, whereas *C. gongota* showed the highest interspecific genetic divergence (24.45%) with *B. lohachata*. In this study, K2P genetic divergence ranged from 3.7% to 24.45% (Figure 4). Ali et al. (2020) showed that K2P genetic divergences were 17% for the Cyprinidae family. Ude et al. (2020) found that intergeneric divergence ranged from 15.80% to 37.00% in their study. Díaz et al. (2016) found a low interspecific divergence ranging between 4.06% and 19.98%. This study showed very low levels of intraspecific genetic diversity and high levels of interspecific genetic divergence. However, there is no universal standard for intraspecific or interspecific boundaries. Ward et al. (2008) concluded that most fish species exhibit low levels of intraspecific variation because COI is a relatively conserved gene in fishes, supporting the findings.

According to the maximum likelihood phylogenetic tree of COI gene sequences, the seven species are divided into distinct clades with high bootstrap support in Figure 6, and specimens within the same species do not converge. Members of the same species are closely clustered because individual samples are categorized in phylogenetic branches based on their taxonomic affinity (Ardura et al. 2010). *B. lohachata* and *B. rostrata* are two recently separated species from the same lineage, showing a very low level of divergence. However, morphologically, *B. rostrata* is considered synonymous with *B. dario*. DNA barcoding reveals the molecular closeness between these two species. *L. guntea* and *L. annandalei* are closely related, and these species have very similar morphological characteristics, where a taxonomic study also indicated that their range of distribution is about the same, but genetically diverse from each other. The maximum likelihood phylogenetic tree constructed from RAG1 gene sequences effectively differentiates between species, with all four species forming distinct clades supported by high bootstrap values. However, the RAG1 region for *B. lohachata, B. rostrata*, and *B. dario* was not amplified, while the other four species were successfully identified. Page et al. (2020) used RAG1 gene analysis to describe a new species of arrow loach. Although all the morphotypes morphologically appeared to be different species, molecular analyses of the COI and RAG1 genes showed they clustered within the same clade with low genetic distance, indicating they belong to the same group. Variations within clades were found in both genes of *L. guntea* , *A. botia* , *B. dario*, and *C. gongota*, which may reflect the phenotypic evolution as those species showed morphological plasticity (Figure 2).

In the ASAP analysis of the COI gene for species delimitation, the identified best partition indicates the presence of seven putative species, corresponding to the lowest ASAP score, which reflects the most suitable partition. Species clustering and delimitation using the ASAP analysis were reported in marine eels (Bhaskar et al. 2021) and toad‐headed Agamas (Solovyeva et al. 2023).

In the haplotype network of this study, a total of 26 haplotypes were found, with 14 distinct haplotypes. The highest haplotypes were identified in specimens of *A. botia* , and most haplotypes resulted from single mutations. Besides *B. lohachata* and *B. rostrata* , other species have geographically isolated specimens separated by unique haplotypes. Species ( *L. guntea* and *A. botia*) that are at LC status show the highest number of haplotypes, indicating a high level of gene flow, while endangered species ( *B. lohachata* and *B. dario* ) exhibit the lowest number of haplotypes indicating low gene flow, and the number of haplotype frequencies decreases or increases with the conservation status in this study. Deng et al. (2024) showed the haplotype network indicated a high level of gene flow in the *Rana hanluica* population. Wakimura et al. (2023) also found a low‐frequency haplotype in the declining *Gnathopogon caerulescens* fish.

During sampling, ambiguous morphology made it difficult to identify species due to the presence of several morphotypes. *B. rostrata* species is morphologically very similar to *B. dario*. The molecular study focused on whether the 16 morphotypes belonging to the seven species represented the same species or not. The species delimitation method and phylogenetic position ensure the species level of collected specimens. Morphology and molecular approaches diminish all taxonomic confusion in the collected specimens.

All seven species in this study exhibit low levels of genetic diversity, particularly *B. lohachata* , *B. dario*, and *B. rostrata* , which are also considered endangered. Overexploitation, water pollution, and habitat drying can reduce loach populations, which in turn decrease diversity. Conservation strategies such as banning habitat drying and fishing gear, along with increasing the number of sanctuaries, may protect loaches from extinction.

## 5 Conclusion

Through applying morphological and molecular methods, seven species of loaches were identified. The morphological differences were associated with the molecular variations observed among these loaches. Genetic variation, phylogenetic position, and haplotype frequencies are critically linked with the conservation status of the identified loaches. To protect the loaches, conservation strategies including artificial breeding and habitat restoration need to be implemented.

## Author Contributions

**Md. Amdadul Haque:** conceptualization (equal), data curation (equal), formal analysis (equal), investigation (equal), methodology (equal), software (equal), visualization (equal), writing – original draft (equal). **Saima Binte Hadi:** data curation (equal), formal analysis (equal), investigation (equal), methodology (equal), visualization (equal), writing – original draft (equal). **Ayesha Akhter Sumona:** data curation (equal), investigation (equal), methodology (equal), validation (equal), visualization (equal). **Jonaira Rashid:** investigation (equal), resources (equal), supervision (equal), validation (equal), writing – review and editing (equal). **Mohd Golam Quader Khan:** conceptualization (equal), resources (equal), supervision (equal), writing – review and editing (equal). **Md. Samsul Alam:** conceptualization (lead), funding acquisition (lead), resources (equal), supervision (equal), writing – review and editing (equal).

## Conflicts of Interest

The authors declare no conflicts of interest.

## Data Availability

The data that support the findings of the study are openly available in the GenBank of the National Center for Biotechnology Information (NCBI: https://www.ncbi.nlm.nih.gov/). The respective GenBank Accession numbers have been shown in Table 2.
